# Fulminant Acute Ascending Hemorrhagic Myelitis Treated with Eculizumab

**DOI:** 10.3389/fneur.2017.00345

**Published:** 2017-07-27

**Authors:** Nang Boe Ohnmar Hsam, Klemens Angstwurm, Sebastian Peters, Kornelius Fuchs, Gerhard Schuierer, Ulrich Bogdahn, Robert Weissert

**Affiliations:** ^1^Department of Neurology, University of Regensburg, Regensburg, Germany

**Keywords:** acute myelopathy, transverse myelitis, eculizumab, autoimmune myelitis, longitudinally extensive transverse myelitis, spinal cord hemorrhage, C5-inhibitor, interleukin-6

## Abstract

We describe an 18-year-old patient who developed back pain, rapidly ascending sensomotory deficits, bladder dysfunction, Lhermitte’s sign, absent abdominal reflexes of all three levels, brisk tendon reflexes, and positive Babinski’s sign. Magnetic resonance imaging of the spinal cord showed a long segment of cervical and thoracic intramedullary signal hyperintensity suggesting a longitudinally extensive transverse myelitis possibly within the course of a fast progressing ascending immune-mediated hemorrhagic myelopathy. Throughout his illness, the patient deteriorated with tetraplegia, cardiac arrest, and respiratory failure although he received, after exclusion of infective causes, therapy with steroids, immunoglobulins, plasmapheresis, and cyclophosphamide. Interestingly, treatment with the C5-inhibitor eculizumab to prevent complement-mediated spinal cord injury achieved an arrest of clinical deterioration. We propose eculizumab as treatment in fast progressive and potentially fatal immune-mediated spinal cord injury. Furthermore, this case raises awareness for the process of clinical decision-making in severe myelopathies.

## Introduction

Dr. Suchett-Kaye of London was the first to introduce the term “acute transverse myelitis” in 1948 as a complication of pneumonia. Earlier cases of “acute myelitis” have been described in association with or after infection and smallpox vaccination (1). Nowadays, transverse myelitis (TM) is known to be a demyelinating disorder also existing as part of an autoimmune CNS [e.g., neuromyelitis optica spectrum disorder (NMOSD), multiple sclerosis] or multisystem disease (e.g., systemic lupus, neurosarcoidosis). TM can be mild or severe, where treatment should be intensified with cyclophosphamide and plasma exchange (2). Fulminant cases have rarely been described in literature. For example, they can present as necrotizing myelitis or ascending hemorrhagic myelitis (3, 4). Unfortunately, most of these cases were fatal (5–7). Our paper describes a patient with a fulminant ascending hemorrhagic longitudinally extensive TM (LETM), who was unresponsive to common treatment strategies, but successfully treated with intensive care, immunosuppression, and eculizumab.

## Background

An 18-year-old Caucasian apprentice carpenter presented with a 1-day history of urinary retention, sensory disturbances of the trunk and lower extremities, and weakness in both legs. The patient has been in his usual state of health until 4 days before admission, when he reportedly had cervical back pain radiating to his whole back.

His past medical history was unremarkable. He had no recent travels, trauma, allergies, and no history of drug abuse.

On examination, the patient was alert, oriented, and had normal vital parameters. Lhermitte’s phenomenon was present, abdominal reflexes were absent, tendon reflexes were brisk, and Babinski’s sign was positive on the right side. Mild motor weakness was present in the legs as well as paresthesia and partial loss of sensation of touch below the level of the umbilicus. Laboratory tests, including blood count and blood levels of electrolytes, were normal. Systemic symptoms of infection were not present.

**Figure 1:**
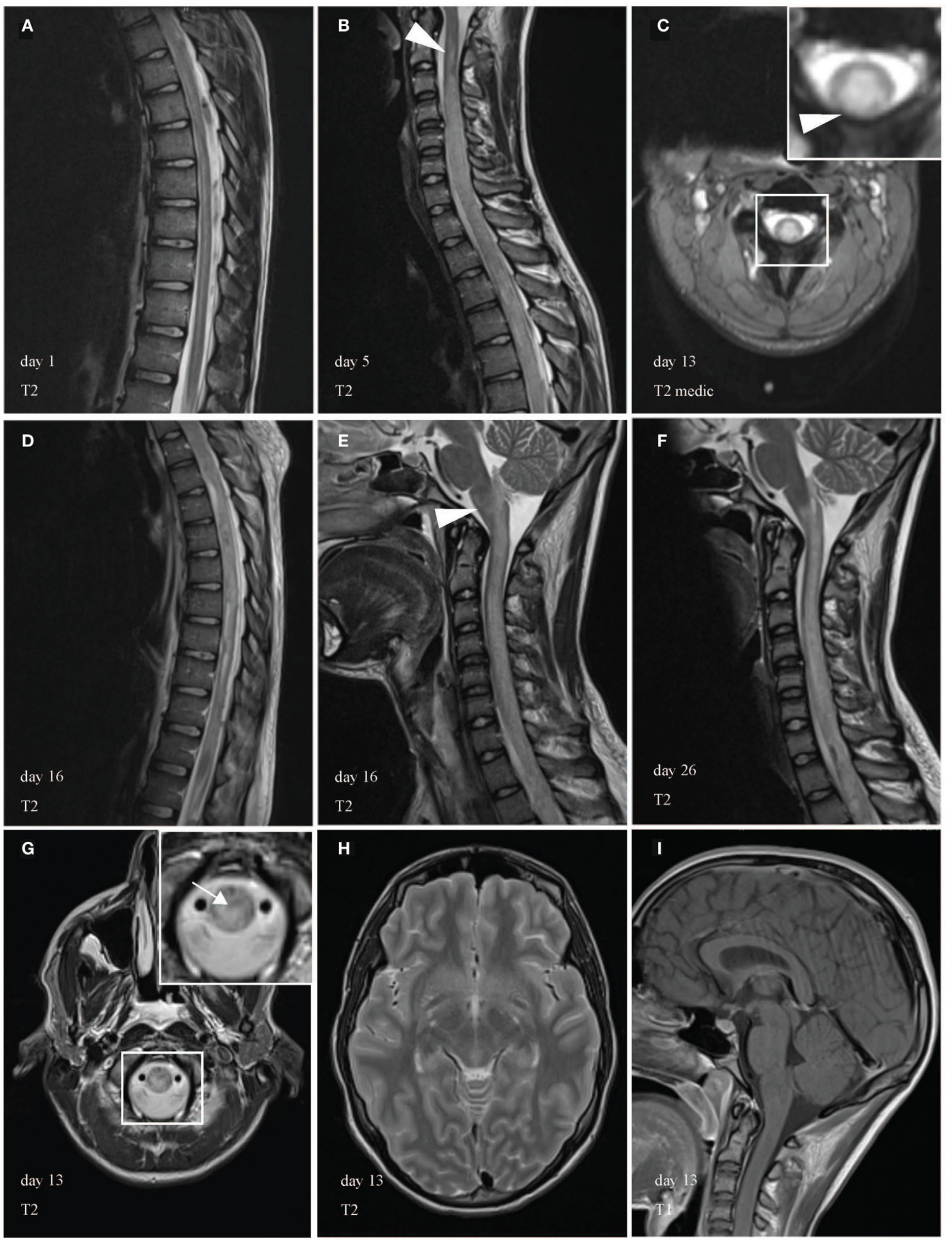
Magnetic resonance imaging (MRI) of the spinal cord during course of illness. MRI showed a longitudinally extensive transverse myelitis (LETM) of the cervical and thoracic spinal cord. In the beginning, the abnormal T_2_-weighted intramedullary signal hyperintensity appeared filigree, extended over more than seven vertebral segments and was most prominent in the anterior gray substance. **(A)** Few days later, MRI revealed cervicothoracic cord swelling a large-scale injury of the cord. During the course of illness, LETM expanded transversally **(C)** and craniocaudally **(B,D)** into the brain stem [**(E,F)**, arrowhead]. **(C,G)** Demonstrate edematous changes in the cervical spinal cord and medulla in axial images (arrow). White box shows area of higher magnification. Brain MRI was unremarkable **(H,I)**.

Magnetic resonance imaging (MRI) of the spine ruled out compression but revealed T_2_-weighted intramedullary signal hyperintensity extending over more than seven vertebral segments (Figure 1) without contrast enhancement in the T_1_-weighted sequences. Initial cerebrospinal fluid (CSF) results were notable for slightly increased leukocytes (25/μl), lactate (2.54 mmol/l), protein (615 mg/l), and albumin (461 mg/l) and no oligoclonal bands (Table S1 in Supplementary Material).

**Figure 2:**
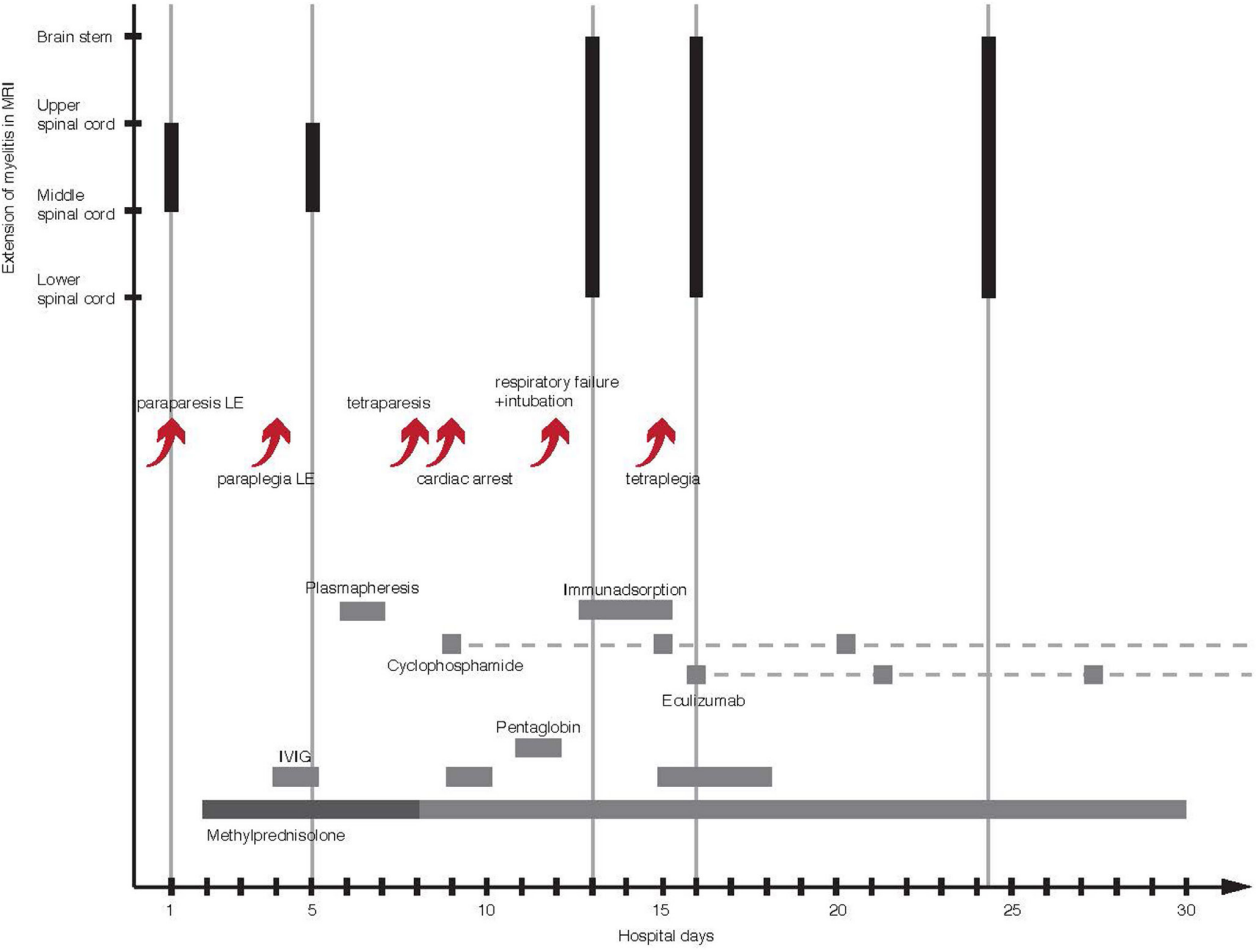
Course of illness with timeline of therapy and craniocaudal extension of LETM in MRI. The arrows show a succession of clinical deterioration and complications in the first 2 weeks of illness. The gray bars represent time and duration of the used treatments. The dashed gray lines mark the continuation of drug administration. The black bars illustrate the ongoing craniocaudal extension of T2 signal hyperintensity. LE, lower extremity; MRI, magnetic resonance imaging; LETM, longitudinally extensive transverse myelitis.

Clinical presentation, extensive MRI cord lesions, inflammatory CSF in contrast to normal laboratory test, most likely suggested myelitis associated with an autoimmune etiology. After treatment with high-dose IV methylprednisolone (sequential treatment regimen is shown in Figure 2), a follow-up lumbar puncture showed normal cell counts with persistent elevation of protein, lactate, and albumin. When paraparesis progressed during steroid therapy, acyclovir and ceftriaxone were given additionally until antibodies and PCR analyses for infective agents were proven negative (Table S2 in Supplementary Material).

Within 4 days, symptoms worsened to paraplegia and onset of paresthesia of hands leading to the addition of IV immunoglobulin (IVIG). Upon clinical and radiological deterioration with a sensory level below T4 and ascending cord lesions with enlargement of the cervical cord, respectively, plasma exchange was started.

The differential diagnosis included myelitis due to post-infectious or post-vaccination reaction, direct infection, systemic autoimmune disease, or vasculitis. In the beginning, an NMOSD was assumed, but serologic analyses for aquaporin-4-IgG (AQP4), myelin oligodendrocyte glycoprotein (MOG) IgG, and visual-evoked potentials were unremarkable. Screening for vasculitis and sarcoidosis parameters and paraneoplastic antibodies in CSF (Table S3 in Supplementary Material) were negative. However, circulating immune complex was slightly increased (116 μl/ml). A brain MRI was normal.

**Figure 3:**
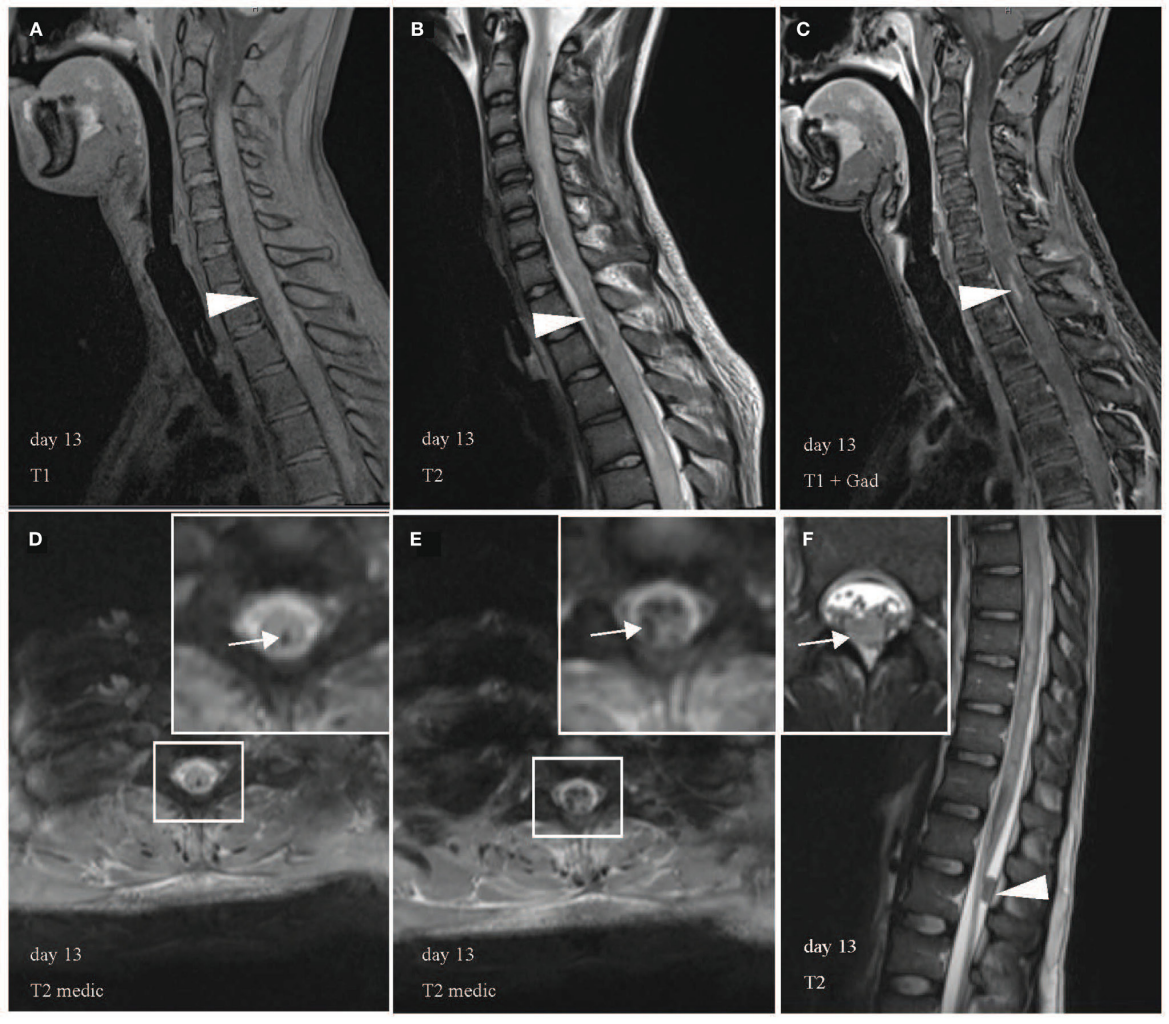
Multiple hemorrhagic lesions in upper and lower spinal cord. Heterogeneous appearance of spinal cord with high and low signal intensity in T1 **(A)**, low signal in T2 **(B)**, and contrast enhancement in T1 with gadolinium **(C)** indicates intramedullary hemorrhage in sagittal images (arrowhead). Representative axial images confirm hemorrhages with hypointense signal abnormalities in T2 medic [**(D,E)**, white arrow]. T2 medic (multi-echo data image combination) is a heavily T2* weighted 2D spoiled gradient echo multi-echo sequence, which is also used to detect hemorrhagic lesions. **(F)** Demonstrates a 1.5 cm × 0.8 cm large hypointense lesion without contrast enhancement in the dorsal spinal canal at the L1 level likely representing a blood clot in sagittal (arrowhead) and axial view (arrow). White boxes display areas of higher magnification.

Tetraparesis developed despite plasma exchange, which had to be paused due to coagulation abnormalities on day 8. The next day, the patient had a cardiac arrest and required a temporary pacemaker. At this time, the patient obviously had a severe case of LETM most likely caused by an aggressive immunological process, which did not respond to common therapeutic options. Consequently, medical therapy was intensified by cyclophosphamide (once a week, 500 mg/m^2^ body surface area), IVIG, and pentaglobin to modulate immunity. Unfortunately, the neurologic symptoms worsened and respiratory failure developed, which necessitated intubation, ventilation, and a percutaneous dilatational tracheostomy. MRI on day 13 revealed further ascending T2 lesion with involvement of the medulla oblongata and hemorrhages in the upper and lower spinal cord (Figures 3A–F). Hypothesizing an autoimmune hemorrhagic myelitis, we escalated therapy by immunoadsorption, followed by IVIG. Nevertheless, symptoms worsened to tetraplegia.

C3 in serum was normal, whereas C4 was initially marginally normal (14.7 mg/dl, normal: >14 mg/dl), but decreased (11.7 mg/dl, normal: 20–40 mg/dl) after about 2 weeks of disease suggesting complement activation.

Challenged by this rapid progressing life-threatening condition, lack of alternative therapies, and the need to stop further tissue destruction to salvage neurologic function, we considered eculizumab (Soliris R), a monoclonal antibody inhibiting the activation of the complement protein C5 and preventing the formation of the membrane attack complex (MAC). After a meningococcal vaccination, 900 mg eculizumab was administered once a week *via* a >30-min intravenous infusion and additional 30 min of observation.

Two days after obtaining eculizumab, the patient recovered from paraplegia of the arms and was again able to bend his elbow (grade 3/5 on the MRC scale for muscle strength). After the third administration, MRI showed no further expansion of lesions (C2-Th11), but a reduced swelling of the cervical cord. On transfer to a paraplegic rehabilitation center on day 30, the patient was ventilated and presented with improved motor and sensory function in his arms. Strength in the legs was 0/5. Regarding the immunosuppressive therapy, steroid therapy was tapered. Cyclophosphamide and eculizumab were given as scheduled for a total course of 4 months. Five months after eculizumab, the patient showed recovered motor function in the arms (5/5, except for hand muscles 4/5), but massive muscle atrophy of hands and legs, paraplegia of legs and anesthesia below C7. The MRI showed a recovery of the medulla and cervical cord, but large areas of atrophy of the thoracic and lumbar spinal cord.

The patient has regularly been seen by the treating physician up to 18 months after the beginning of the disease. He is doing well. He is using a wheel chair without motor support by himself. At the most recent visit, the patient showed further recovered motor function in the arms (5/5, except for hand muscles 4.5/5). The musculature of the arms has regained in diameter and strength. There is some persistent, but compared to earlier visits, slightly reduced atrophy of hands of C8 innervated musculature. There is persistent paraplegia of the legs and anesthesia below C8. He is in high spirits and is planning to start an apprenticeship.

## Methods

Electrochemiluminescence (MesoScale Discovery^®^, MD, USA) was performed with serum samples from healthy controls (*n* = 11) and the patient (pretreatment and posttreatment with iv steroids) and with a pretreatment CSF sample of the patient. V-PLEX Human Biomarker 40-Plex Kit (MesoScale Discovery^®^) was used according to manufacturer’s instructions.

## Results

Multiplex analysis revealed, relative to control, elevated serum interleukin-6 (IL-6), IL-8, IL-15, angiogenesis marker (Flt-1, vascular endothelial growth factor), and vascular injury marker (serum amyloid A, C-reactive protein, vascular cell adhesion protein-1) levels, which returned to baseline after treatment with iv steroids (Table 1). Serum IL-7, IL-17A, eotaxin, eotaxin-3, and phosphatidylinositol-glycan biosynthesis class F protein levels remained increased after steroids, whereas IL-12/-23p, Tie-2, and intercellular adhesion protein-1 were higher only after steroid treatment. Accordingly, CSF IL-6 (988.98 pg/ml) and IL-8 (308.14 pg/ml) seemed to be markedly higher. Earlier findings revealed that healthy patients or patients with diseases other than TM showed much lower IL-6 in CSF within a range of 2.5 ± 0.72 to 5.5 ± 2.4 pg/ml (8–10) and IL-8 (2.21 ± 1.87 to 29.6 ± 8.4 pg/ml) (9, 10), respectively.

**Table 1:** Results of the electrochemiluminescence analysis of serum and CSF.

	Marker	Ctrl serum (pg/ml)	Pt. serum 1 (pg/ml)	Pt. serum 2 (pg/ml)	Pt. CSF (pg/ml)
Proinflammatory panel	TNF-alpha	1.7	1.52	2.03	1.63
	**IL-8**	12.15	34.42^+^	8.74	308.14
	**IL-6**	0.72	2.96^+^	0.93	988.98
	IL-4	0	0	0	1.14
	IL-2	0	0	0	0
	IL-1β	0	0	0	0
	IL-13	0	0	0	0.65
	IL-12p70	0	0.32	1.16	2.22
	IL-10	0.32	0.44	0.29	2.41
	IFN-gamma	13.12	1.5	2.16	0.9

Cytokine panel	TNF-β	0.14	0.38	0.88	0.15
	**IL-7**	16.16	71.36^+^	62.81^+^	4.93
	**IL-17A**	1.14	10.92^+^	2.73^+^	2.37
	IL-16	280.51	160.94	153.33	18.41
	**IL-15**	2.43	4.31^+^	2.22	4.9
	**IL-12/IL-23p**	85.45	19.68	217^+^	5.97
	GM-CSF	0.12	0.06	0.25	0.2

Chemokine panel	TARC	153.88	167.93	93.48	24.72
	MIP-1β	107.08	177.36	127.67	22.79
	MIP-1alpha	0	15.34	13.64	43.18
	MDC	973.06	204.18	897.7	140.37
	MCP-4	139.8	142.69	130.5	8.57
	**MCP-1**	280.28	126.73	124.71	1,248.2
	IP-10	120.78	192.75	173.97	0
	**Eotaxin-3**	18.52	40.9^+^	30.29^+^	13.16
	**Eotaxin**	142	234.51^+^	209.27^+^	34.93

Angiogenesis panel	**VEGF**	162	404.42^+^	263.14	3.62
	VEGF-C	631.53	580.61	869.86	0
	VEGF-D	1,286.66	890.36	536.98	26.47
	**Tie-2**	2,755.81	2,491.62	3,560.68^+^	0
	**Flt-1**	98.7	166.07^+^	80.23	54.91
	**PIGF**	28.38	53.24^+^	41.66^+^	60.83
	bFGF	14.1	2.74	2.82	0.34

Vascular injury panel	**SAA**	1,455,578.15	131,682,134.1^++^	3,511,136.25	731,605.91
	**CRP**	1,501,929.29	6,867,529.02^+^	2,539,092.28	651,735.84
	**VCAM-1**	419,338.49	1,163,126.37^++^	718,949.46	3,593,598.73
	**ICAM-1**	195,632.20	259,640.21	532,294.00^++^	1,731,257.65

*CSF, cerebrospinal fluid; Ctrl, control; Pt, patient; bFGF, basic fibroblast growth factor; CRP, C-reactive protein; Flt-1, fsm-like tyrosine kinase-1; GM-CSF, granulocyte–macrophage colony-stimulating factor; ICAM-1, intercellular adhesion protein-1; IFN-gamma, interferon gamma; IL, interleukin; IP-10, interferon gamma-induced protein 10; MCP, macrophage chemoattractant protein; MDC, human macrophage-derived chemokine; MIP, macrophage inflammatory protein; PIGF, phosphatidylinositol-glycan biosynthesis class F protein; SAA, serum amyloid A; TARC, thymus and activation regulated chemokine; Tie-2, angiopoetin-1; TNF, tumor necrosis factor; VCAM-1, vascular cell adhesion protein-1; VEGF, vascular endothelial growth factor. ^+^ and ^++^ show elevated and markedly elevated levels. The bold font indicates that for highlighted parameters enhanced values were measured in serum*.

## Discussion

This case highlights that eculizumab in combination with immunoadsorption and cyclophosphamide is successful in severe immune-mediated hemorrhagic TM to stop further cord damage.

Longitudinally extensive TM is a disabling disorder described as a spinal cord T2 signal hyperintensity that extends over at least three vertebral segments (11, 12). Although often associated with neuromyelitis optica, there is a broad list of differential diagnosis of LETM with an acute/subacute presentation including idiopathic, inflammatory, neoplastic, infectious, and vascular causes (12, 13). Due to the hemorrhages in the spinal cord, the diagnostic criteria for typical AQP4-IgG seronegative NMOSD were not fulfilled for this patient (14). Anti-MOG antibodies, which are often seen in AQP4 negative NMO, were also not present. In addition, initially the patient did not respond to the immune therapeutic approaches, which normally influence NMOSD. However, raised levels of proinflammatory IL-6 and IL-8 among other parameters in serum and CSF in our patient strongly suggested an autoimmune myelitis. IL-6 is a potent inducer of Th17 cells and the observed increased levels of IL-17A in blood could be explained by the heightened IL-6 level in blood and CSF (15). IL-6 has been reported to be significantly elevated in CSF of TM patients and to be necessary to promote spinal cord damage, e.g., demyelination and axonal injury. Furthermore, increased levels were shown to correlate with clinical disability (8). The complement system forms a central part of the innate immunity by eliminating pathogens, but complement activation can also have a proinflammatory role and is important in inflammatory diseases and tissues damage (16). Its effectivity is achieved by formation of the MAC. Recent findings proposed that sub-lytic MAC might promote inflammation by inducing IL-6 and IL-8 (17).

The intensified therapeutic approach with the terminal complement inhibitor eculizumab attained clinical stabilization and improvement. This immunotherapy was well tolerated except for mild fever, which was often observed after treatment with eculizumab. Nevertheless, the patient developed visual hallucinations and anxiety secondary to the meningococcal prophylaxis with ciprofloxacin. After a change of the regimen to ceftriaxone, no other complications occurred. The patient improved much clinically. From the status that he was tetraplegic, he was then again able to use his arms. Beside this noteworthy result, the spinal cord injury ultimately stabilized and was reduced as seen in the MRI. Additionally, azathioprine was given as preventive therapy after finishing treatment with cyclophosphamide and eculizumab.

Since eculizumab has been approved by the US Food and Drug Administration for the treatment of paroxysmal nocturnal hemoglobinuria and atypical hemolytic uremic syndrome (18), current studies are investigating its efficacy in neurologic diseases such as myasthenia gravis and NMOSD (19, 20).

Longitudinally extensive TM can rarely represent spinal cord neoplasms, but since our lesions showed no gadolinium enhancements as seen in almost all intramedullary metastases (21), we considered a neoplastic cause to be very unlikely. We did not obtain a cord biopsy due to ethical reasons and the danger of aggravating the disease further.

It is noteworthy that eculizumab comes with very high costs and therefore accessibility to this medicine may be difficult. So far, no alternative complement inhibitor with lower cost is available on the market.

In patients like ours, in which IL-6 levels in the CSF and blood are found increased early in the course of disease, monoclonal antibodies targeting IL-6 (e.g., siltuximab and sarilumab) or the IL-6 receptor [e.g., tocilizumab (22)] could also be considered as a therapeutic approach to prevent severe tissue damage.

## Conclusion

In conclusion, though rare and prognosis of fulminant myelitis is poor, additional treatment with a complement inhibitor may be beneficial for serious cases to minimize poor outcome with severe lifelong disability and secondary complications, which place high emotional and economic worries on affected individuals and their relatives.

## Ethics Statement

This study was carried out in accordance with the recommendations of the Ethics Committee of the University of Regensburg. The reported patient has given written informed consent in accordance with the Declaration of Helsinki.

## Author Contributions

NH and RW analyzed the data and drafted the manuscript, figures, and tables. KA, KF, GS, and UB analyzed data. SP performed electrochemiluminescence and contributed to analysis of the data.

## Conflict of Interest Statement

RW reports personal fees from Roche, Merck Serono, Biogen, Genzyme, and Novartis as well as grants from Novartis and Sanofi Aventis outside the submitted work. The other authors have nothing to disclose.
