# Supplementary material for: Fulminant Acute Ascending Hemorrhagic Myelitis Treated with Eculizumab
Source: Front Neurol. 2017 Jul 27;8:345. doi: 10.3389/fneur.2017.00345 (PMC5529383; doi:10.3389/fneur.2017.00345)
Supplement: Supplementary file 1 [file Table_1.PDF]

Supplementary table 1: Results of analysis of cerebrospinal fluid.

Variable	Normal range	Initial lumbar puncture	1 day after IV steroids
White-cell count	0-5 (cells/µl)	25*	0.33
Protein level	150-450 (mg/l)	615*	76,4*
Lactate	1,11-2,45 (mmol/l)	2,54*	3,32*
Albumin	110-350 (mg/l)	461*	430*
Alb./quotient L/S	<5,2	9,26	10,21
Oligoclonal bands	negative	negative	-
Intrathecal IgG/IgA/IgM	no	no	no
