# Supplementary material for: Fulminant Acute Ascending Hemorrhagic Myelitis Treated with Eculizumab
Source: Front Neurol. 2017 Jul 27;8:345. doi: 10.3389/fneur.2017.00345 (PMC5529383; doi:10.3389/fneur.2017.00345)
Supplement: Supplementary file 2 [file Table_2.PDF]

Supplementary table 2: Infective agents tested in serum and CSF.

Agent	Blood	CSF	CSF PCR
Treponema pallidum	negative		
Human T-lymphotropic virus 1/2	negative		
Measles IgG	positive	negative	
Measles IgM	negative	negative	
Mumps IgG	positive	negative	
Mumps IgM	negative	negative	
Anti-Herpes simplex virus 1/2 IgG	negative	negative	
Anti-Herpes simplex virus 1/2 IgM	negative	negative	
Anti-Varicella zoster virus IgG	positive	negative	
Anti-Varicella zoster virus IgM	marginal	negative	
Anti-Epstein–Barr virus IgM Immunoblot	negative		
Anti-Tick-borne encephalitis virus IgG	negative	negative	
Anti-Tick-borne encephalitis virus IgM	negative	negative	
Anti-*Borrelia burgdorferi* IgG ELISA	positive	negative	
Anti-*Borrelia burgdorferi* IgM ELISA	marginal	negative	
Anti-*Borrelia burgdorferi* IgG Immunoblot	positive		
Anti-*Borrelia burgdorferi* IgM Immunoblot	negative		
Anti-*Mycoplasma* IgG	negative		
Anti-*Mycoplasma* IgM	negative		
Anti-hepatitis A virus	positive		
Anti-hepatitis B core	positive		
Anti-hepatitis B surface	positive		
Hepatitis B surface antigen	negative		
Anti-hepatitis C virus	negative		
Human immunodeficiency virus	negative		
Anti-*Bordetella pertussis* IgG	positive		
Anti-*Bordetella pertussis* IgA	positive		
Anti-*Bordetella pertussis* IgM	negative		
Bartonella henselae			negative
Enterovirus			negative
Echovirus			negative
Poliovirus			negative
Coxsackievirus			negative
Cytomegalovirus			negative
Human herpes virus-6			negative
Legionella pneumoniae/spp.			negative
JC polyomavirus			negative
Herpes simplex virus 1/2			negative
Varicella zoster virus			negative
Epstein–Barr virus			negative
