# Supplementary material for: Fulminant Acute Ascending Hemorrhagic Myelitis Treated with Eculizumab
Source: Front Neurol. 2017 Jul 27;8:345. doi: 10.3389/fneur.2017.00345 (PMC5529383; doi:10.3389/fneur.2017.00345)
Supplement: Supplementary file 3 [file Table_3.PDF]

Supplementary table 3: Paraneoplastic antibody panel tested in serum and CSF

**Antibody name**	**Serum/CSF**
Anti-Hu	negative
Anti-Ri	negative
Anti-Yo	negative
Anti-Tr	negative
Anti-myelin	negative
Anti-Ma/Ta	negative
Anti-GAD65	negative
Anti-amphiphysin	negative
Anti-aquaporin 4	negative
Anti-glutamate receptor	negative
Anti-GABAB receptor	negative
Anti-LGl1	negative
Anti-CASPR2	negative
Anti-NMDA receptor	negative
Anti-glycine receptor	negative
